# 

**Published:** 1891-09

**Authors:** 


### TABLE OF CONTENTS.

ORIGINAL COMMUNICATIONS. PAGE

The Bacteria of the Air as a Disturbing Factor in Dental and Surgical Operations. By W. D. Miller, M.D., D.D.S., Berlin, Germany . . . 581

Growth of the Cementum. By R. R. Andrews, D.D.S., Cambridge, Mass. 587

Dental Infirmary Patients—The Use and Abuse of Dental Charity. By Richard Grady, M.D., D.D.S., Baltimore, Md. . . . . . . . . . . . 592

Dental Responsibility for Early Diagnosis of Tumors of the Mouth and Jaws. By Arthur K. Stone, A.M., M.D. . . . . . . . . . . . . . 602

A Comparison of the Methods and Surroundings of English and American Dentists. By Waldo E. Boardman, D.M.D., Boston, Mass. . . . . 609

Observations on Dental Caries. By Dr. A. M. Ross, Springfield, Mass. . 612

Tincture of Chloride of Iron. By Thomas J. Giblin, D.M.D. . . . . . 614

The Relations and Resources of our Specialty. By Horatio C. Meriam, D.M.D., Salem, Mass. . . . . . . . . . . . . . . . . . . . . . . . 617

REPORTS OF SOCIETY MEETINGS.

American Medical Association . . . . . . . . . . . . . . . . . . . . . 627

American Academy of Dental Science . . . . . . . . . . . . . . . . . . 631

Harvard Odontological Society . . . . . . . . . . . . . . . . . . . . . 640

National Association of Dental Faculties . . . . . . . . . . . . . . . 649

National Association of Dental Examiners . . . . . . . . . . . . . . . 652

EDITORIAL.

American Dental Association . . . . . . . . . . . . . . . . . . . . . . 656

The National Association of Dental Faculties and the National Association of Dental Examiners . . . . . . . . . . . . . . . . . . . . . . 657

OBITUARY.

D. Frank Whitten, D.M.D. . . . . . . . . . . . . . . . . . . . . . . . 659

Resolutions of Respect on the late Dr. William H. Atkinson, of New York 660

DOMESTIC CORRESPONDENCE.

Errata . . . . . . . . . . . . . . . . . . . . . . . . . . . . . . . . . 660

